# RETRACTION: Lithium and Copper Induce the Osteogenesis‐Angiogenesis Coupling of Bone Marrow Mesenchymal Stem Cells via Crosstalk between Canonical Wnt and HIF‐1α Signaling Pathways

**DOI:** 10.1155/sci/9765145

**Published:** 2026-07-27

**Authors:** Stem Cells International

RETRACTION: Z. Tan, B. Zhou, J. Zheng, et al., “Lithium and Copper Induce the Osteogenesis‐Angiogenesis Coupling of Bone Marrow Mesenchymal Stem Cells via Crosstalk between Canonical Wnt and HIF‐1α Signaling Pathways,” *Stem Cells International* 2021, no. 1 (2021): https://doi.org/10.1155/2021/6662164.

The above article, published online on 8 March 2021 in Wiley Online Library (wileyonlinelibrary.com), has been retracted by agreement between the Journal Editor‐in‐Chief, Renke Li, and John Wiley & Sons Ltd. The retraction has been agreed after an investigation of image concerns.

Initially, the authors requested a correction to Figures 1a–c and 6a, after stating that they had repeated the experiments. However, image concerns involving Figure 6a were raised by *Actinopolyspora biskrensis* on PubPeer [1]. Specifically, the top left panel, “Alizarin red staining −25 μM Cu^2+^”, in Figure 6a shares unexpected similarities with the bottom left panel, “CPP–Mineralized nodule”, in Figure 4a of an article by different authors [2].

During the journal’s investigation, the following additional concerns were noted:

– The bottom left corner of Figure 1a shares unexpected similarities with the top right panel, “Alizarin red staining–Co‐stimulation”, in Figure 6a;
– Figure 2c (3) and 2c (4) share unexpected similarities, with Figure 2c (4) appearing to be a magnification of Figure 2c (3);
– Figure 6a also shares unexpected similarities between the top left panel, “Alizarin red staining −25 μM Cu^2+^” and the top middle panel, “Alizarin red staining −500 μM Li^+^”;
– Three Western blot bands for GADPH in the far‐right panel of Figure 4d appear to be duplicated in the far‐right panel of Figure 6c, after vertical flip, with the figures describing completely different experimental conditions.

The authors believe that they had sufficiently indicated that Figure 2c (4) is a magnification of Figure 2c (3), however, the editor disagrees. The authors also stated that images which appeared in the earlier article [2] had been stolen from an unsecured, shared computer, but they could not provide their original raw data due to the length of time since the experiments were originally conducted. As such, the editor can no longer consider the results to be reliable. Therefore, the article must be retracted.

The authors disagree with this retraction.
